# Ectopically expressing *MdPIP1;3*, an aquaporin gene, increased fruit size and enhanced drought tolerance of transgenic tomatoes

**DOI:** 10.1186/s12870-017-1212-2

**Published:** 2017-12-19

**Authors:** Lin Wang, Qing-Tian Li, Qiong Lei, Chao Feng, Xiaodong Zheng, Fangfang Zhou, Lingzi Li, Xuan Liu, Zhi Wang, Jin Kong

**Affiliations:** 1 College of Horticulture, China Agricultural University, Beijing, China; 2 College of Biological Sciences, China Agricultural University, Beijing, China

**Keywords:** Aquaporin, MdPIP1;3, Water transportation, Fruit size, Drought tolerance, Stomata closure

## Abstract

**Background:**

Water deficit severely reduces apple growth and production, is detrimental to fruit quality and size. This problem is exacerbated as global warming is implicated in producing more severe drought stress. Thus water-efficiency has becomes the major target for apple breeding. A desired apple tree can absorb and transport water efficiently, which not only confers improved drought tolerance, but also guarantees fruit size for higher income returns. Aquaporins, as water channels, control water transportation across membranes and can regulate water flow by changing their amount and activity. The exploration of molecular mechanism of water efficiency and the gene wealth will pave a way for molecular breeding of drought tolerant apple tree.

**Results:**

In the current study, we screened out a drought inducible aquaporin gene *MdPIP1;3*, which specifically enhanced its expression during fruit expansion in ‘Fuji’ apple (*Malus domestica* Borkh. cv. Red Fuji). It localized on plasma membranes and belonged to PIP1 subfamily. The tolerance to drought stress enhanced in transgenic tomato plants ectopically expressing *MdPIP1;3*, showing that the rate of losing water in isolated transgenic leaves was slower than wild type, and stomata of transgenic plants closed sensitively to respond to drought compared with wild type. Besides, length and diameter of transgenic tomato fruits increased faster than wild type, and in final, fruit sizes and fresh weights of transgenic tomatoes were bigger than wild type. Specially, in cell levels, fruit cell size from transgenic tomatoes was larger than wild type, showing that cell number per mm^2^ in transgenic fruits was less than wild type.

**Conclusions:**

Altogether, ectopically expressing *MdPIP1;3* enhanced drought tolerance of transgenic tomatoes partially via reduced water loss controlled by stomata closure in leaves. In addition, the transgenic tomato fruits are larger and heavier with larger cells via more efficient water transportation across membranes. Our research will contribute to apple production, by engineering apples with big fruits via efficient water transportation when well watered and enhanced drought tolerance in transgenic apples under water deficit.

**Electronic supplementary material:**

The online version of this article (10.1186/s12870-017-1212-2) contains supplementary material, which is available to authorized users.

## Background

Water shortage, which challenges many apple orchards worldwide, severely limits apple growth and production. In particular, the fruits size, the important factor in attracting the consumer and determining price, entirely depends on water absorption and transportation during fruit expansion. In maintaining a water supply, increased input raises costs and reduces profit. Therefore, the gene wealth and the underlying molecular mechanism are explored for the molecular breeding of water-efficient apple cultivar.

Water transportation across cell membranes is entirely regulated by amount and activity of water channels, known as aquaporins. Most of them are located in plasma membranes and tonoplast membranes to transport water in or out of the cells or vacuoles [1–4]. Aquaporins at root epidermal and inner cells, vascular tissues of stems, leaves and their adjacent cells, form a main continuous system for water uptake and transportation. The amount of aquaporins is very high and forms nearly 10-15% of plasma membrane or tonoplast proteins [5–7], suggesting the reliance of plants on their routine function. Aquaporins belong to the major intrinsic protein (MIP) family. In this large family, many different members are located at nearly all kinds of tissues and cells to participate in different physiological processes including water uptake and transportation, stomatal behavior, fruit development and ripening, and the response to various environmental stressors [1, 8–20].

Because of the sessile nature of plants, they were unavoidably subjected to different environmental stressors, such as high salinity, drought and extreme temperature. These abiotic stresses directly result in difficulty of water uptake in plants. Whether the plants can survive in the stresses or conversely undergo irreversible wilting and subsequent dying, largely depends on the efficient work of aquaporins. In the tolerant plants, aquaporin genes can be induced to provide more water channels. The post-translational modification confers higher activity of aquaporins for better regulation. In addition, the aquaporins regulated by the stress hormone ABA, function in guard cells to close stomata, which cut water loss. Taken together, more efficient water absorption and transportation and less water loss allow the plants to keep good water balance even under stresses. But if the aquaporins cannot orchestrate stress resistance, crops suffer from stresses, which lead to unavoidable yield loss. Various aquaporins play different roles in the water absorption and usage. Therefore overexpression of some aquaporins conferred the transgenic plants enhanced plant resistance to water deficiency under osmotic stresses or subjection to extreme temperatures, while some other aquaportions conferred decreased stress tolerance [21–23].

Fruit growth is divided into distinct developmental stages, including an intense cell division period, a cell expansion period followed by a ripening period [24]. In apple, fruit cell expansion has two peak stages, one from 60 days to 100 days and the other from 120 days to 160 days after anthesis [25]. Cell division strongly affects the final fruit size in many species, but cell expansion also results in the change of fruit size [26–31]. With cell wall relaxation and loosening, cell turgor and water potential decrease, and water enters to the fruit cells via xylem and phloem tissues to stimulate cell expansion [32]. In the expansion stage of fruit development, the quick water transportation into the fruit cells makes length and diameter increase rapidly, which partially decides the final fruit size. Accumulated research has reported the high expression of aquaporins during fruit development, suggesting their possible active water transportation for fruit expansion [15, 20, 33]. Furthermore, the increased expression of *FaPIPs* and possible enhanced subsequent water flow through the plasma membranes, were proved to be in good correlation with firmness [13]. Taken together, the aquaporins might be involved in fruit development, but their roles in determining fruit expansion through water transportation across cell membranes remains unclear.

Herein, in order to isolate the gene for efficient water transportation and maintaining, which can increase fruit size when well watered, and improve drought tolerance under water deficit, we screened out a drought inducible, fruit-expansion-inducible aquaporin gene *MdPIP1;3* in the ‘Fuji’ apple (*Malus domestica* Borkh. cv. Red Fuji). Its ectopic expression conferred transgenic tomatoes with enhanced drought tolerance via decreased water loss, controlled by guard cells expansion and subsequent stomata closure. In addition, the transgenic tomatoes have a bigger berry with larger cells when well watered. Our research paves a way to engineer apples with big fruits when well watered and also enhanced drought tolerance under water deficit, which will contribute to apple production.

## Methods

### Plant materials and growth conditions

‘Micro-Tom’ tomato (*Solanum lycopersicum* L.) seeds were sterilized with 75% alcohol for 5 min, then 4% sodium hypochlorite for 12 min, and washed 6 times with sterile water. After being sowed on Murashige and Skoog (MS) medium, the seeds were grown in darkness at 22 ± 2 °C for 3-4 days, and then transferred to a growth chamber of 16 h light (~150 μEm^−2^ s^−1^) /8 h dark at 22 ± 2 °C. One-month-old plants were transferred into soil in greenhouse.

The apple seeds (*Malus baccata* Borkh.) were sown in vermiculite in a growth chamber with light intensity of 100 μmol m^−2^ s^−1^ at 22 ± 2 °C. After three weeks, the seedlings were watered with complete nutrient solution.

The *Arabidopsis* (Colombia 0) seeds were sterilized and sowed on MS medium according to Wang et al. (2015) [34]. The seedlings were grown vertically in a growth chamber with 16 h light (~150 μEm^−2^ s^−1^) /8 h dark at 22 ± 2 °C. After 7-10 days, the seedlings were transplanted into soil for the following protoplast extraction.

### The expression of *MdPIP1;3* in drought treatment and fruit development

*Malus baccata.* Seedlings, which reached 5-6 cm high were transferred into the nutrient solution containing 20% PEG for drought treatment, and sampled at 0, 4, 8, 12, 24 h, 3d and 5d, respectively. The expression of *MdPIP1;3* gene in leaves was detected by semi-quantitative RT-PCR. The semi-quantitative RT-PCRs were performed as follows: pre-cycling step of 94 °C for 5 min, which was followed by 32 cycles of 94 °C for 30s, 55 °C for 40s, and 72 °C for 40s, and then a final extension at 72 °C for 10 min. The PCR system (20 μL) contained 10 μL of 2 × ES Taq MasterMix, 2 μL of cDNA, 6 μL of ddH_2_O, 0.5 μM forward primer and reverse primer, respectively. The PCR products (5 μL) were electrophoresed in a 1% agarose gel. Gene-specific primers for PCR amplification were designed using Primer Premier 5 software (Premier, Vancouver, Canada) (Table 1). The 257-bp fragment of *MdActin* (XM_008393049.2) was selected as control. Semi-quantitative RT-PCR experiment was repeated in triplicate.

**Table 1:** The sequences of primers in the construction

The name of primer	The sequences of primer
MdPIP1;3-F	5’-GGATCCATGGAGGGCAAGGAAGAAG-3’
MdPIP1;3-R	5’-TCTAGATTAGCTCCTGGACTTGAAAGG-3’
MdPIP1;3-GFP-F	5’-GAATTCATGGAGGGCAAGGAAGAAG-3’
MdPIP1;3-GFP-R	5’-GGATCCGCTCCTGGACTTGAAAGGAA-3’
MdPIP1;3-RT-F	5’-TGATGCCAAGAGGAATGCC-3’
MdPIP1;3-RT-R	5’-CACGCTTGGGATGACCACT-3’
MdActin-F	5’-CAATGTGCCTGCCATGTATG-3’
MdActin-R	5’-CCAGCAGCTTCCATTCCAAT-3’
SlActin-F	5’-CCACGAGACCACATACAACT-3’
SlActin-R	5’-TGAGGGAAGCCAAGATAGAG-3’
SlPIP1;2-RT-F	TTTCACTCACTAACTCCCATCAAT
SlPIP1;2-RT-R	TAAAGAAAGAGGAAAGTAGCCACA
SlPIP1;3-RT-F	ACCATCAAATAATCATCAGAGCA
SlPIP1;3-RT-R	AGGATAAAATAAAAATTATTTTCAT
SlPIP1;5-RT-F	ATGATTATGCCAAGGGAGATGA
SlPIP1;5-RT-R	GCCAAATGAACCAAGAACACAG
SlPIP2;1-RT-F	ACGTACCCGTGTTGGCACCTCTTCC
SlPIP2;1-RT-R	ATGTTCGTCCCACGCCTTGTCACC
SlPIP2;5-RT-F	GTCCTCTTCCAGCCATCCA
SlPIP2;5-RT-R	ACCACTGAGCACAATGTTACCG
SlPIP2;7-RT-F	ATTCCCATATCCCTGTGTTGGCTCC
SlPIP2;7-RT-R	AGCTGCAGCTCTCAAAATGTATTGG

During the fruit development, ‘Red Fuji’ apple fruits (*Malus domestica* Borkh. cv. Red Fuji) were collected in the ‘Beiliu’ orchard (40°10’ N, 116°4′ E) of ‘Liu’ town, Changping District, Beijing, China. The apple fruits were sampled at different stages of fruit development from June 4 to October 15, 2012. The sampling interval was 2 weeks for the first nine sampling points, and then the interval was shortened to 1 week for the last three sampling points. At each time point, five apples were collected, frozen in liquid nitrogen immediately and stored at −80 °C. Total RNA extraction and cDNA synthesis were conducted according to Wang et al. (2015) [34]. Subsequent semi-quantitative RT-PCR was carried out to detect the expression of *MdPIP1;3* during fruit development. The experiment was repeated in triplicate.

### Isolation and bioinformatics analysis of *MdPIP1;3* gene

The complete open reading frame (ORF) of the *MdPIP1;3* genes, which was 861 bp, was amplified with the specific primers using the cDNA of ‘Fuji’ apple as template. All the primers were designed by Primer Premier 5.0 software and shown in Table 1. The PCR reaction was carried out using the following cycles: 94 °C for 3 min; 30 cycles at 94 °C for 30s, 52 °C for 30s, and 72 °C for 50s; and 72 °C for 10 min. After sequencing, the sequence of *MdPIP1;3* was uploaded to the NCBI (GenBank: KY952167).

The bioinformatics analysis was performed according to Wang et al. (2015) [34], to analyze the conserved functional domain, molecular weight and isoelectric point of MdPIP1;3. Multiple alignments and phylogenetic tree were conducted by DNAMAN software (LynnonBiosoft, San Ramon, California, USA), and MEGA5.2 software (MEGA Team, Tempe, Arizona, USA) by neighbor-joining method and a bootstrap test with 1000 iterations, respectively, which used the amino acid sequences of homologous proteins of MdPIP1;3: AtPIP1;4 (Arabidopsis, AEE81879.1), SoPIP1;2 (spinach, AAR23268.1), ZmPIP1;2 (maize, NP_001104934.1), OsPIP1;3 (rice, Q9SXF8.2) and PcPIP1;1 (pear, BAB40142.1).

### The subcellular localization of MdPIP1;3

To observe the subcellular localization of MdPIP1;3, its coding sequences, without stop codon, was amplified using the primers with *Eco*RI/*Bam*HI (Table 1) by PCR as described above. The sequenced PCR product was subcloned into the transient expression vector pEZS-NL with an eGFP (enhanced Green Fluorescent Protein) for the fusion protein MdPIP1;3-GFP.

In the transient expression system of *Arabidopsis* protoplasts, the *MdPIP1;3-eGFP* fusion plasmid and the *eGFP* vector plasmid were introduced into protoplasts via PEG-mediated transformation, respectively, as described by Wang et al. (2015) [34]. The *eGFP* plasmid was used as control. The subcellular localization of MdPIP1;3 was observed by confocal microscopy (Nikon, Japan) with 600× magnification (60 × 10) after transient expression for 16-24 h.

### Generation of transgenic micro-tom plants ectopically expressing *MdPIP1;3*

In order to explore in vivo functions of MdPIP1;3 in drought response and fruit development, the transgenic tomato plants ectopically expressing *MdPIP1;3* were generated. The *MdPIP1;3* gene was cloned into the plant expression vector pBIN438 to construct pBIN438-*MdPIP1;3* plasmid, which was introduced into *Agrobacterium tumefaciens* strain EHA105 via freeze-thaw method [35]. Transgenic tomato plants ectopically expressing *MdPIP1;3* were obtained using EHA105 carrying pBIN438-*35 s*::*MdPIP1;3* by *Agrobacterium tumefaciens*-mediated transformation [36]. The wild type plants that regenerated from untransformed cotyledons were taken as control.

To detect the expression level of *MdPIP1;3* in transgenic tomato plants, semi-quantitative RT-PCRs were conducted as described above and repeated three times. Gene-specific primers for PCR amplification were designed using Primer Premier 5 software (Table 1). The 250-bp fragment of *SlActin* (U60478.1) was selected as control. The transgenic plants confirmed by PCR were planted in greenhouse with a natural light cycle. The collection of transgenic tomato fruits and seeds and the separation of foreign genes were conducted according to Wang et al. (2014) [36]. Each gene had three independent homozygous T2 generation lines with high gene expression which were selected for further study.

### Drought treatment, dehydration rate measurement and leaf stomata observation

For drought treatment, the transgenic tomato plants ectopically expressing *MdPIP1;3* and the wild type plants weren’t watered in greenhouse. After 20 days without watering, the soil volumetric moisture content (*ϴ*_V_) dropped from 43 to 45% (well-watered condition) to 0.5-1% (water deficit stress), the plants were re-watered every two days until the *ϴ*_V_ increased to 43-44% to detect their recovery capability. The *ϴ*_V_ was detected by a Soil Moisture Sentor (NC®, Beijing; Type: SU-LB). The phenotype was observed and recorded using photography. The experiment was repeated three times.

40-mm (in length) leaves of transgenic lines ectopically expressing *MdPIP1;3* and wild type plants were detached, placed on the filter paper for 15 h at room temperature (20 ± 2 °C) for an in vitro dehydration rate experiment. The weight of leaves was measured. The mass ratio, as an indicator of dehydration rate, was calculated by the weight after drought treatment divided by the original weight before treatment. The experiment was repeated three times.

Five leaves were collected from transgenic and wild type plants under normal condition and drought treatment, respectively. Leaf stomata observation was performed using a TM3000 microscope (HITACHI, Tokyo, Japan). Stomata were observed randomly in 50 visual sections of the same area [37]. The experiment was repeated in triplicate.

### Effects of the ectopic expression of *MdPIP1;3* on the expression of other *SlPIP1s* and *SlPIP2s* under drought stress

The wild type and three transgenic tomato lines ectopically expressing *MdPIP1;3* were germinated on MS medium under dark for 3-4 days at 22 ± 2 °C, which were transferred to a growth chamber of 16 h light (~150 μEm-2 s-1) /8 h dark at 22 ± 2 °C for growth. Then the seedlings with four leaves were transferred carefully to MS medium with 300 mM mannitol solution for drought treatment. The seedlings were sampled at 0 h and 4 h, respectively, for RNA extraction and semi-quantitative RT-PCR as described above.

To confirm whether ectopically expressing *MdPIP1;3* influence other *PIP1s* and *PIP2s* in tomato plants, three *SlPIP1s* and three *SlPIP2s* were selected as candidate genes according to the previous research [38], including *SlPIP1;2* (Solyc03g096290.2.1), *SlPIP1;3* (Solyc08g008050.2.1), *SlIP1;5* (Solyc01g103270.2.1) and *SlPIP2;1* (Solyc06g011350.2.1), *SlPIP2;5* (Solyc02g083510.2.1), *SlPIP2;7* (Solyc01g111660.2.1). The sequences of primers were in Table 1. The experiment was repeated three times.

### Tomato fruit development analysis between transgenic and wild type plants

To estimate the influence of the exogenous aquaporin genes insertion on the fruit development, transgenic and wild type lines (containing 3-5 plants per line) were grown in the greenhouse with a natural light cycle. From the beginning of the flowering, ten buds per plant were selected and marked. Then the lengths and the diameters of these tomato fruits from the transgenic and the wild type plants were recorded at 10, 20, 25, 30, 35, 40, 50, 60 and 70 days after flowering, respectively. The experiment was repeated in triplicate.

### Fruit size and fresh weight of transgenic and wild type tomato fruits

To further analyze the differences between transgenic and wild type fruits, the fruit size and the fresh weight of each 25 mature fruits from all the three transgenic lines and wild type were measured. The epidermis of a total of 10 mature fruits from each three individual transgenic line and the wild type, were cut into 4 × 4 mm pieces. These epidermis pieces were frozen at ultralow temperature (−196 °C), coated by gold, and observed under ultralow temperature condition with 300× magnification by cryo-electron microscopy (HITACHI, Japan). A total of 6-8 pieces of each fruit were observed and photographed. The cell number per mm^2^ was counted in these pictures to ensure that at least 500 cells for each individual fruit and 5000 cells for each transgenic line were counted. The experiment was repeated three times.

### Statistical analysis

The data were expressed as mean ± SD. One-way ANOVA was used for normality evaluation followed by Tukey-Kramer multiple comparison test by SPSS software (IBM, Armonk, NY, USA), with differences considered significant if P<0.05 or very significant if *P* < 0.01.

## Results

### *MdPIP1;3* enhanced its expression under drought treatment and at the expansion period during apple development

**Fig. 1:**
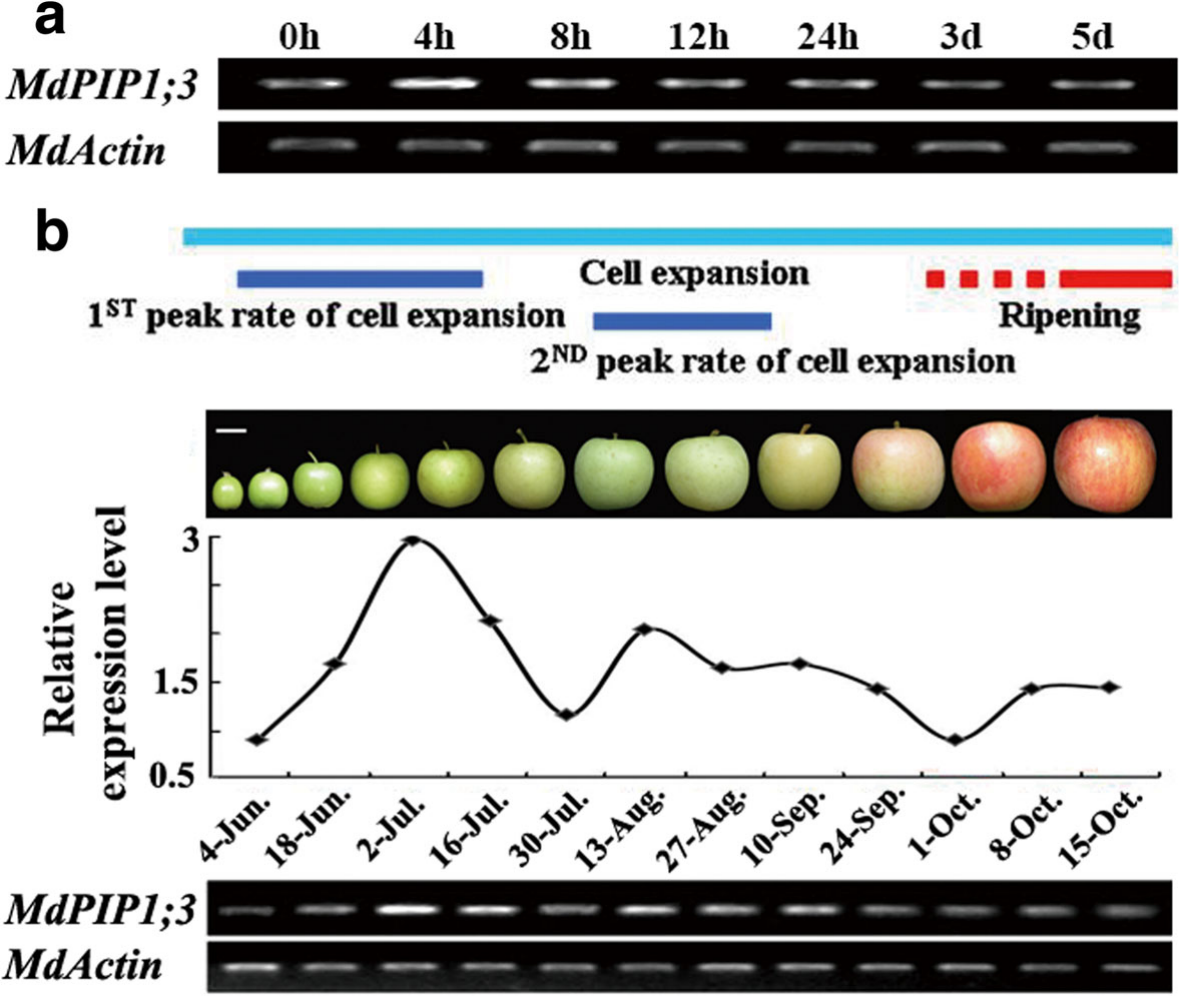
*MdPIP1;3* expression in drought treatment and during apple fruit development. **a** *MdPIP1;3* expression in drought treatment at different time points. The apple seedlings were subjected to drought treatment (nutrient solution containing 20% PEG) and sampled at 0, 4, 8, 12, 24 h, 3d and 5d, respectively. **b** *MdPIP1;3* expression during apple fruit development. The apple fruits were sampled at different stages of fruit development from June 4 to October 15. Gene expression of *MdPIP1;3* was detected by semi-quantitive RT-PCR and *MdActin* was as control. The semi-quantitive RT-PCR experiments were repeated three times

The *MdPIP1;3* expression was induced at 4 h after drought treatment in apple leaves (Fig. 1a). During the apple development, the *MdPIP1;3* was expressed differently. Interestingly, the two *MdPIP1;3* expression peaks were in accord with the two cell expansion periods (from June to July and from August to September) (Fig. 1b), suggesting that the MdPIP1;3 might be involved in the drought response and the fruit development of apple, especially at expansion period of fruits.

### MdPIP1;3 is an aquaporin of PIP1 subfamily

**Fig. 2:**
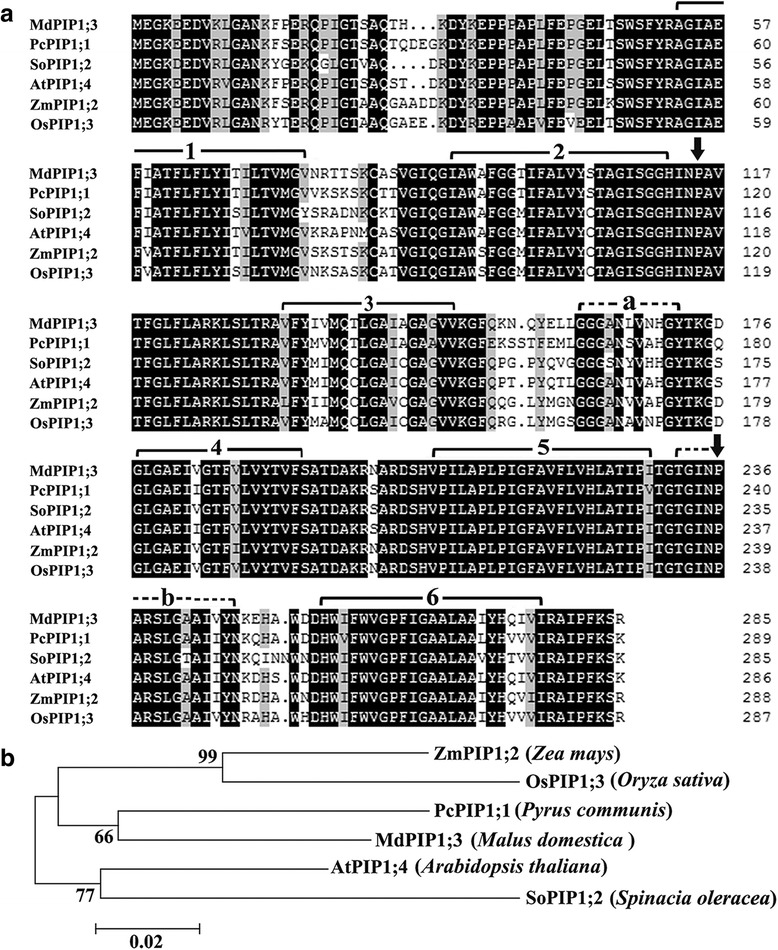
Multiple alignments and the phylogenetic tree of MdPIP1;3. **a** Multiple alignments of MdPIP1;3. The black color highlights the same sequence, and the gray color identifies the sequence with only one amino acid difference. The solid lines with numbers, the arrows and the dash lines with lowercases show the transmembrane domains, the conserved NPA domains (Asn-Pro-Ala) and the signature sequences of plasma membrane water channel protein family, respectively. **b** The phylogenetic tree of MdPIP1;3. The GenBank accession numbers are MdPIP1;3 (KY952167), AtPIP1;4 (AEE81879.1), SoPIP1;2 (AAR23268.1), ZmPIP1;2 (NP_001104934.1), OsPIP1;3 (Q9SXF8.2) and PcPIP1;1 (BAB40142.1). The phylogenetic tree was constructed using the neighbor-joining method and a bootstrap test with 1000 iterations, using MEGA5.2 software

The bioinformatics analysis predicted that MdPIP1;3, has 286 amino acids with relative molecular mass of 30.72kD and isoelectric point of 9.23. The multiple alignments showed that MdPIP1;3 has six transmembrane domains, two conserved NPA domains (Asn-Pro-Ala) and signature sequences of plasma membrane water channel protein family (GGGANXXXXGY and TGI/TNPARSL/FGAAI/VI/VF/YN) shared by other PIP1s, which are typical features of aquaporins. It has a high similarity of 83-89% in amino acid sequences with these homologous PIP1s (Fig. 2a). Furthermore, the phylogenetic tree showed that MdPIP1;3 is clustered with the PIP1 subfamily and has closer relationship with PcPIP1;1 in pear, compared with other PIP1s. It suggests MdPIP1;3 is a member of aquaporin, belonging to the PIP1 subfamily (Fig. 2b).

### MdPIP1;3 is localized on the plasma membrane

**Fig. 3:**
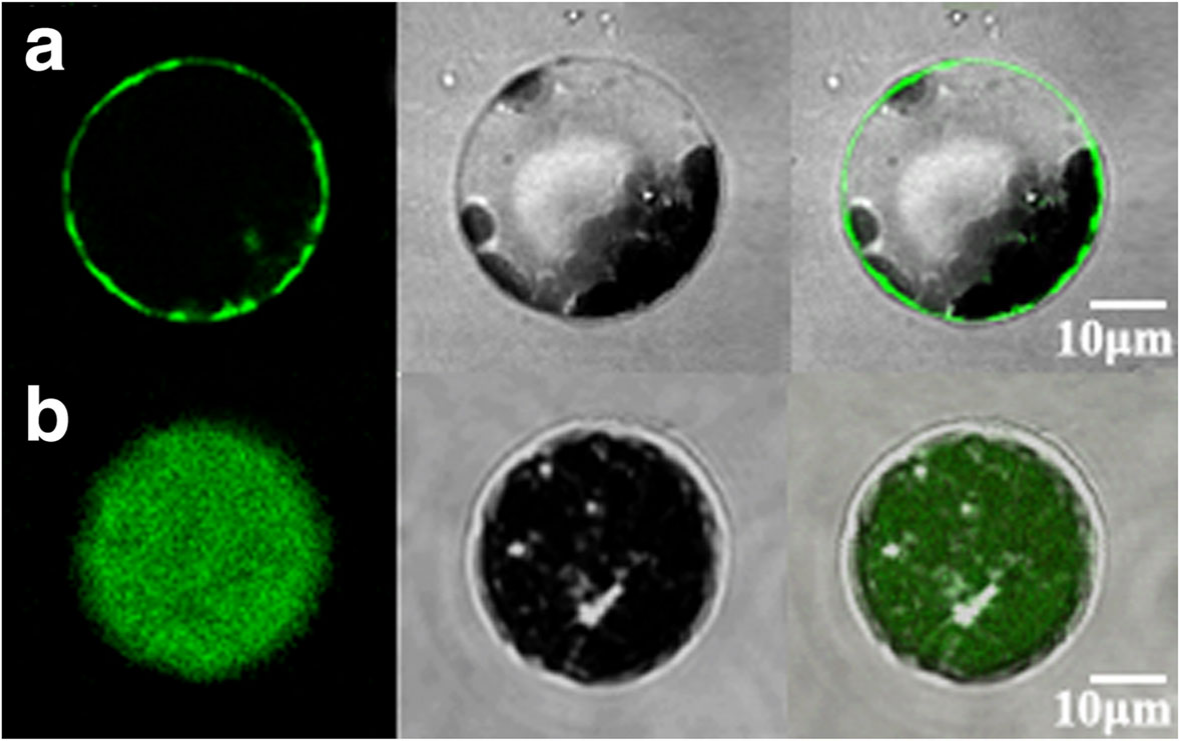
Subcellular localization of MdPIP1;3 on plasma membrane. **a** Transgenic *Arabidopsis* protoplasts transiently expressing MdPIP1;3-eGFP fusion protein. **b** Transgenic *Arabidopsis* protoplasts transiently expressing eGFP control. They were observed using confocal microscopy with 600 × magnification (objective magnification × eyepiece magnification = 60 × 10). Left: Green fluorescence images; Middle: The bright-field images; Right: The merged fluorescent images. Bar, 10 μm

In order to determinate the subcellular localization of MdPIP1;3, the *MdPIP1;3*-*eGFP* plasmid and the *eGFP* control plasmids were transiently expressed in the *Arabidopsis* protoplasts, which were observed using confocal microscopy. The results showed that MdPIP1; 3-eGFP was localized on the plasma membrane (Fig. 3a), while the eGFP control was observed throughout whole protoplast (Fig. 3b).

### Ectopically expressing *MdPIP1;3* in transgenic tomato plants

**Fig. 4:**
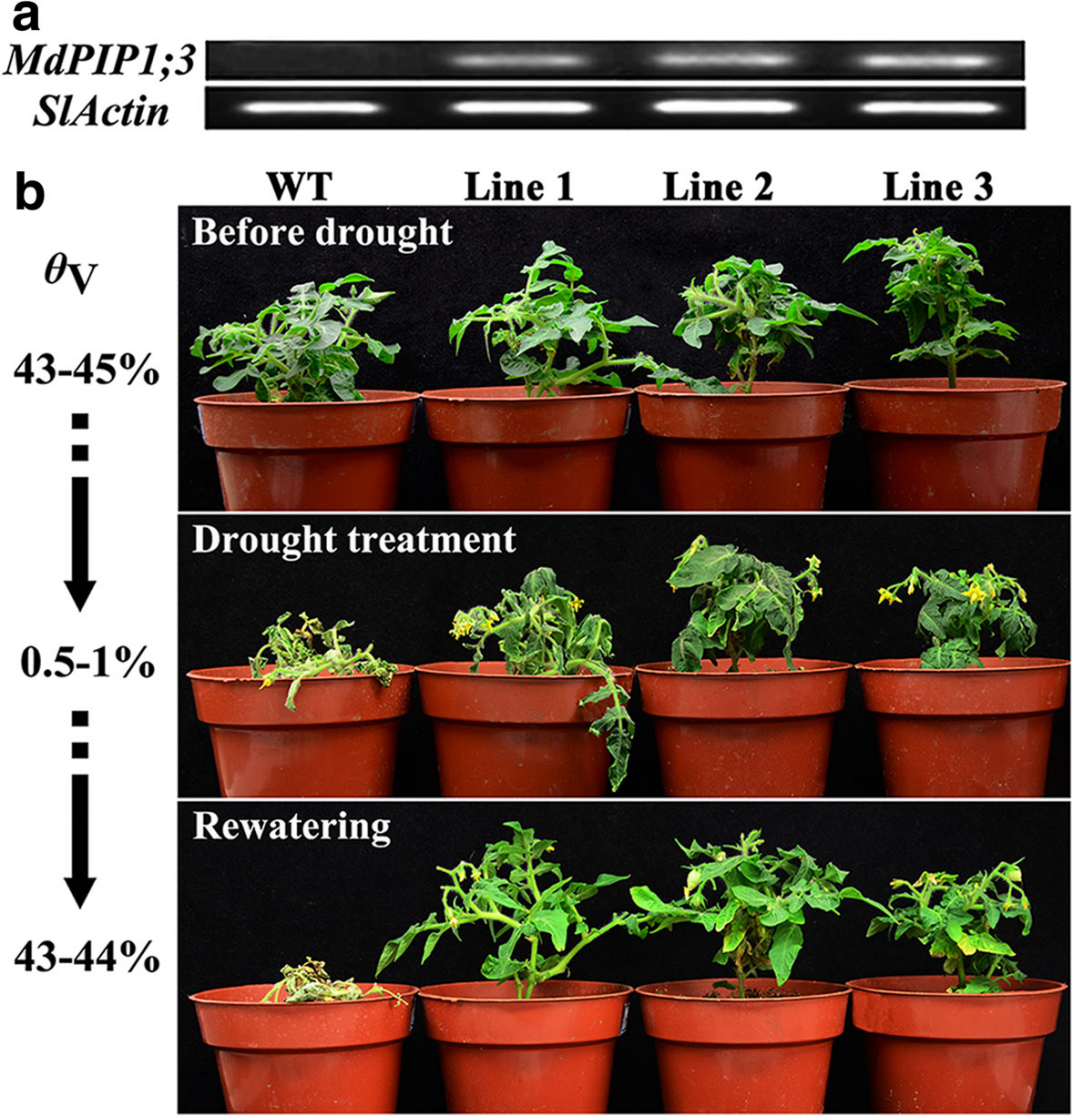
Phenotype of transgenic tomato plants ectopically expressing *MdPIP1;3* under drought stress. **a** Expression of *MdPIP1;3* in the three transgenic lines and wild type by semi-quantitative RT-PCR, with the *SlActin* as internal gene. **b** Growth condition of three transgenic tomato lines and wild type under drought stress. *ϴ*_V_: the soil volumetric moisture content. The experiments were repeated in triplicate

In order to investigate the in vivo roles of *MdPIP1;3*, *Agrobacterium tumefaciens*-mediated transformation was applied to generate transgenic tomato plants ectopically expressing *MdPIP1;3,* which is selected by kanamycin resistance and confirmed by PCR. According to the result of expression levels of *MdPIP1;3* tested by RT-PCR, three independent T2 lines were selected for further phenotyping (Fig. 4a).

### Transgenic tomato plants ectopically expressing *MdPIP1;3* showed enhanced tolerance to drought stress

**Fig. 5:**
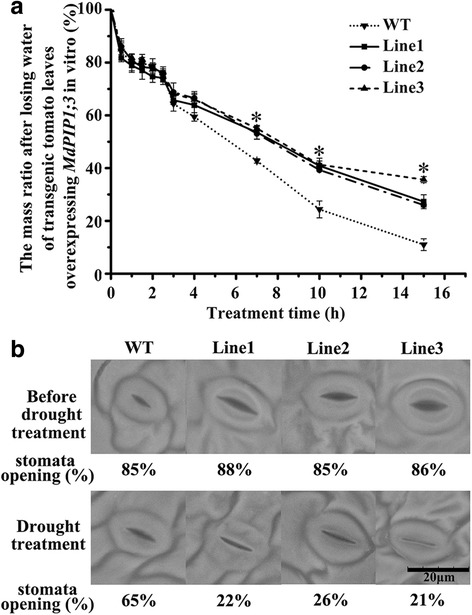
In vitro dehydration rate and the leaves stomata observation. **a** Dehydration rate experiment of detached leaves (40-mm in length) was conducted for 15 h at room temperature (20 ± 2 °C) to measure the weight of leaves. The mass ratio was calculated by the weight after drought treatment divided by the original weight before treatment. **b** Stomata of leaves under drought stress were observed using the TM3000 microscope. Stomata were observed randomly in 50 visual sections with the same area. The experiments were repeated three times

All of the three T2 generation independent transgenic lines ectopically expressing *MdPIP1;3* were subjected to drought stress. Before drought treatment, the transgenic and the wild type plants grew well similarly. After drought treatment for 20 days, the three transgenic lines showed slight wilting, while the wild type plants wilted and etiolated more severely. When re-watering these plants, the transgenic plants recovered quickly, but the wild type plants died (Fig. 4b), suggesting that the transgenic tomato plants had enhanced tolerance to drought stress. In addition, the detached leaves in transgenic and wild type plants had basically the same dehydration rate during the first three hours. While from the beginning of the forth hour, the wild type leaves lost water faster than the transgenic leaves (Fig. 5a).

To further study how ectopically expressing *MdPIP1;3* in tomatoes release water more efficiently, we observed how the stomata behave in both transgenic and wild type plants before and after drought, respectively. Interestingly, the stomatal aperture in transgenic plants was bigger than the wild type at normal growth condition, while after drought treatment for 12 days, most of the guard cells were engorged and the stomata of 77% closed in the three transgenic lines, but the stomata of approximately 65% in wild type plants were still open (Fig. 5b), indicating the stomata closure in transgenic lines responded to drought stress more quickly than the wild type.

In order to detect the effect of ectopically expressed *MdPIP1;3* on expression of other *SlPIP1s* and *SlPIP2s* under drought stress in transgenic tomato plants, three *SlPIP1s* and three *SlPIP2s* possibly involved in drought response were selected for semi-quantitative RT-PCR analysis. The results showed that all the six genes enhanced their expressions under drought stress. But there weren’t any expression difference between wild type and transgenic lines either under water deficit or normal condition (Additional file 1: Figure S1). It indicated that ectopically expressed *MdPIP1;3* doesn’t affect the expression of other *SlPIP1s* and *SlPIP2s* under drought stress.

### Transgenic tomatoes ectopically expressing *MdPIP1;3* grew faster than wild type during the expansion period

**Fig. 6:**
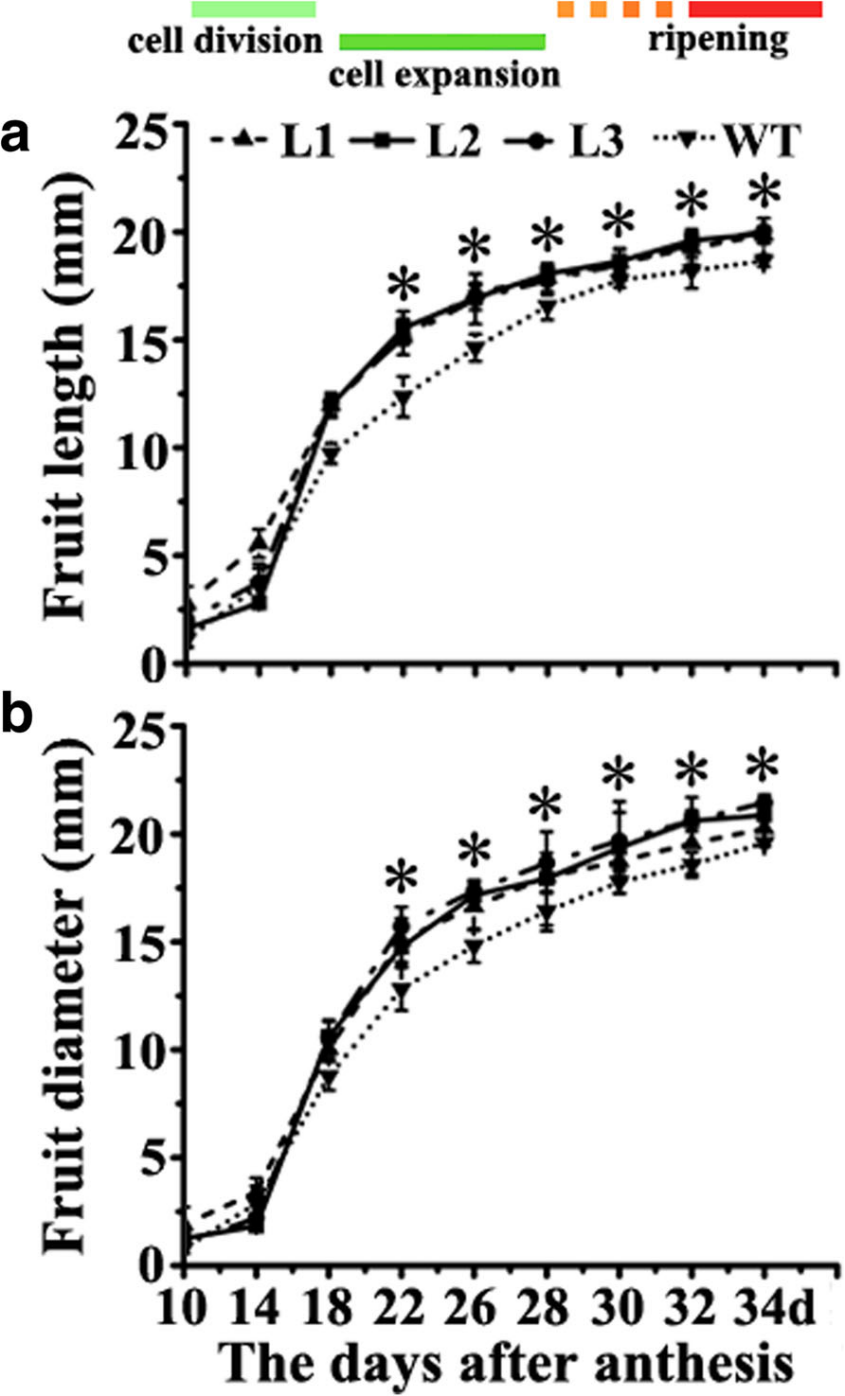
Growth curves of fruit length and diameter in transgenic and wild type tomatoes. **a** Curve of fruit length. **b** Curve of fruit diameter. The lengths and the diameters of tomato fruits from transgenic and wild type tomatoes were recorded at 10, 20, 25, 30, 35, 40, 50, 60 and 70 days after flowering, respectively. The experiment was repeated in triplicate

To investigate how MdPIP1;3 affected fruit development, the whole fruit development process after anthesis was observed. The lengths and the diameters of transgenic and wild type fruits were recorded to compare their growth rate. The results showed that in the expansion period, the slopes of curves in transgenic tomatoes were more sharp than the wild type (Fig. 6), suggesting that the transgenic tomatoes grew faster than the wild type during the expansion stage.

### Transgenic tomatoes ectopically expressing *MdPIP1;3* were bigger and heavier than the wild type

**Fig. 7:**
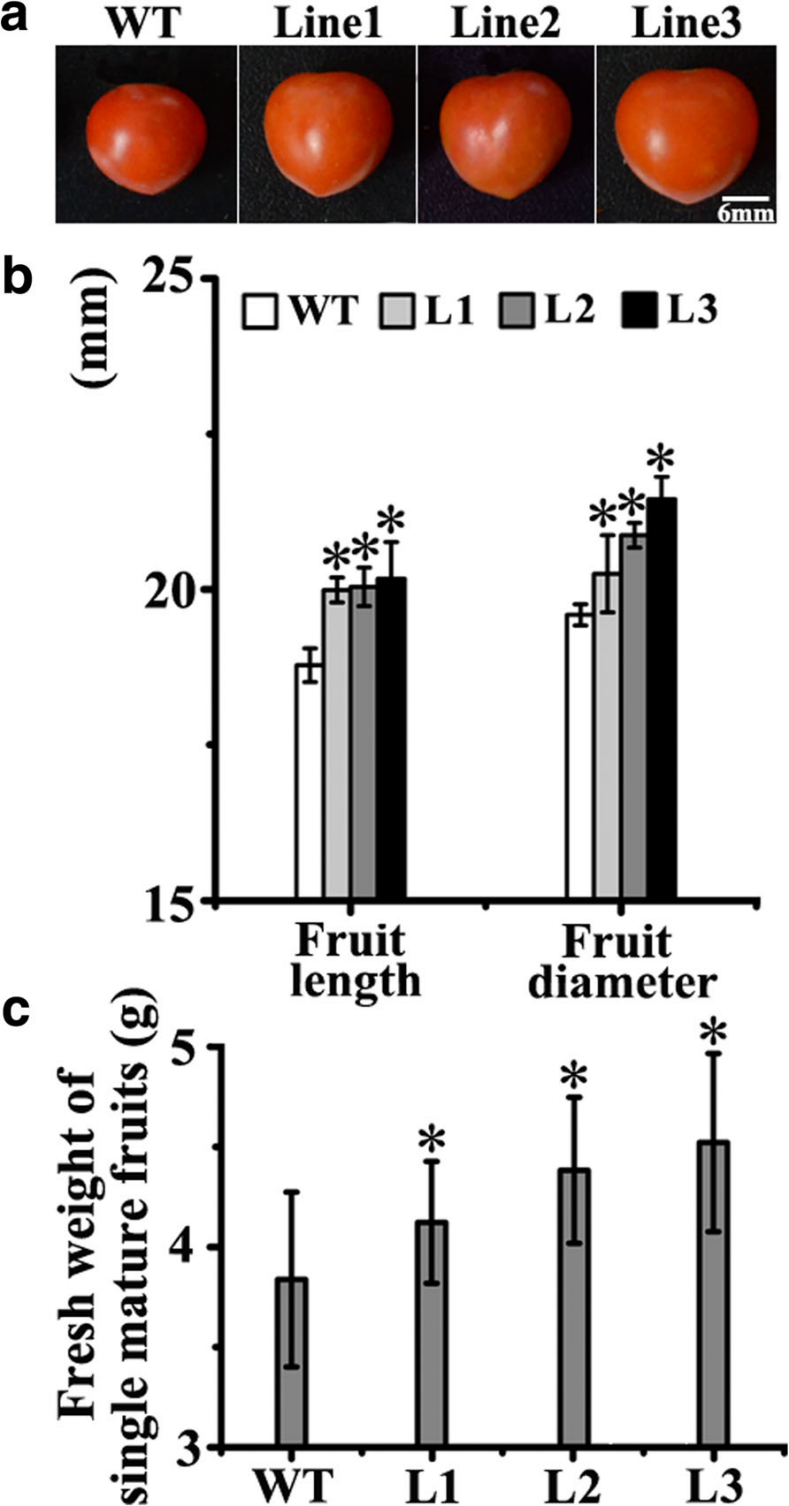
The fruit size and the fresh weight of transgenic and wild type mature tomatoes. **a** The mature fruits of transgenic and wild type tomatoes. Bar, 6 mm. **b** The fruit length and the fruit diameter of mature fruits. **c** The fresh weight of mature fruits. The experiment was repeated three times

**Fig. 8:**
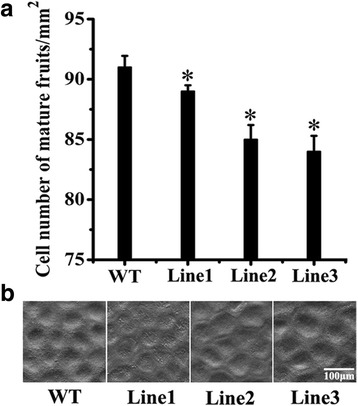
Fruit cells size of transgenic and wild type mature tomatoes. **a** Fruit cells number per mm^2^. **b** Fruit cell size. The epidermis were cut into 4 × 4 mm pieces, which were frozen in ultralow temperature (−196 °C), coated by gold, and observed under ultralow temperature condition with 300× magnification by cryo-electron microscopy. The cell number per mm^2^ was counted to ensure that at least 500 cells for each individual fruit and 5000 cells for each transgenic line were counted. Bar, 100 μm. The experiment was repeated three times

The results showed that the transgenic fruits were bigger than the wild type (Fig. 7a and b). The cell number per mm^2^ in transgenic fruits was less than the wild type, inferring the cell size of transgenic tomatoes was larger than the wild type (Fig. 8). Besides, the fresh weights of transgenic fruits were also heavier than the wild type (Fig. 7c). This data indicated that the aquaporin might be involved in fruit development by controlling more water transportation into fruit cells.

## Discussion

Global warming is expected to increase the frequency of drought stress. This represents a challenge to apple producers all over world. Under drought stress, inputs necessary to maintain orchards will increase dramatically. Compared with the traditional crossing and selection, the breeding time for drought-tolerant apple either selected by marker-assistant selection or by GM technology will shorten. Aquaporins, in charge of the transmembrane water transportation, were reported to be involved in both water deficiency response and fruit development. The efficient transmembrane water transportation can improve water absorption, transportation and maintaining. Therefore, it seems possible to engineer plants with big fruits via efficient water transportation when well watered, and improved drought tolerance by increasing the expression level of aquaporin genes.

Herein, a gene encoding for aquaporin localized in plasm membrane, *MdPIP1;3* gene, was screened out because of its enhanced expression both under drought stress and during expansion stages of apple fruit development (Fig. 1), suggesting its possible roles in drought response and fruit development through regulating water flow. Bioinformatics research proved that MdPIP1;3 has the signature domains of AQP (GGGANXXXXGY and TGI/TNPARSL/FGAAI/VI/VF/YN), and two NPA (Asn-Pro-Ala) conserved domains forming the hydrophilic channel of aquaporins (Fig. 2a). Furthermore, the results of phylogenetic tree and subcellular localization showed that MdPIP1;3 was clustered with the PIP1 subfamily and localized on the plasma membrane (Figs. 2b and 3), inferring its function as a plasma membrane intrinsic protein, aquaporin.

As aquaporin is a big gene family, only some members are involved in the response to water deficit [39]. In the current study, a drought-inducible aquaporin gene, *MdPIP1;3* was selected. In order to observe the in vivo effects of MdPIP1;3 on drought tolerance, it was introduced to the model plant ‘Micro-Tom’ tomato by *Agrobacterium tumefaciens*-mediated transformation. The ectopically expressed *MdPIP1;3* gene conferred the transgenic tomato enhanced drought resistance. From 20-day-drought treatment to re-watering, the leaves of transgenic tomato plants experienced slight wilting and restored to normal growth quickly, but the wild type plants wilted and died even after re-watering (Fig. 4b). The further semi-quantitative RT-PCR of other aquaporin genes possibly involved in drought tolerance [38], proved that the enhanced drought tolerance of transgenic tomato completely resulted from the ectopically expressed *MdPIP1;3* (Additional file 1: Figure S1). In addition, the results from the detached leaves showed that three transgenic lines lost water significantly slower than the wild type (Fig. 5a), inferring MdPIP1;3 functions to hold water in the leaves of transgenic plants. Further research found that the stomata of transgenic leaves closed quickly, while that of the wild type remained open when the soil volumetric moisture content dropped from 43 to 45% to 0.5-1% (Fig. 5b). It suggested that the enhanced drought tolerant might partially result from decreased water loss under stress. It is well-known that a pair of guard cells controls the stomata closure. When water enters into guard cells to make them engorged, the stomata were closed. Under drought stress, it seems that more abundant MdPIP1;3 allows more rapid guard cell engorgement in transgenic leaves and therefore results in reduced water loss through stomata. This can explain transgenic tomato plants that hold more water under drought treatment. Similarly, *NtAQP1* and *VfPIP1* changed the stomatal conductance of transgenic *Arabidopsis* under salt and drought stresses [14, 17, 19]. Hence, our results implied that ectopically expressing *MdPIP1;3* might confer transgenic plants drought tolerance by efficient stomata closure and decreased water loss through transpiration, which at least partially depends on quicker guard cell engorgement under water deficit.

Although based on the correlations between expression of aquaporin genes and morphological observations, there were reports on the involvement of aquaporins in fruits development, suggesting their possible role in facilitating water redistribution within the growing berry, there still isn’t any in vivo functional evidence [33, 40, 41]. To explore the function of *MdPIP1;3* in fruit development in vivo, the fruit size was recorded during fruit development in wild type and transgenic tomato plants ectopically expressing *MdPIP1;3*. By comparison of transgenic and wild type tomato fruits during the expansion period, the slopes of both fruit length and diameter curves in transgenic tomatoes were more sharp than wild type, suggesting that the transgenic fruits expanded faster (Fig. 6). Actually, the final fruit sizes of transgenic tomatoes were also significantly larger than wild type. In addition, both length and diameter of transgenic fruits was significantly higher than wild type (Fig. 7a and b). Accordingly, transgenic tomatoes were significantly heavier than wild type (Fig. 7c). By counting nearly a thousand cells from tomato fruit skin of every transgenic line and control, there were clearly less cells in the transgenic plants per mm^2^ than wild type (Fig. 8), indicating fruit cells of transgenic plants were bigger than wild type. The correlation of fruit cell size, fruit size and fruit weight has been proved in the previous research [42]. Therefore we can conclude that the larger cells result in bigger tomato fruits of transgenic plants. The high levels of MdPIP1;3 might transport more water into the fruit cells, making these cells engorged to a larger size and finally giving a bigger and heavier berry. Although the expression of *AQP* genes in fruit development have been reported in banana, tomato, apple and grape [15, 20, 33, 43], there are no reports of its roles in fruit expansion and fruit size decision. For the first time, we proved that MdPIP1;3 increased both fruit size and fresh weight of transgenic tomatoes by transporting more water into cells under well-watered condition. Taken together, MdPIP1;3 might contribute to an engineered apple with larger size when well watered, and enhanced drought tolerance whilst also cutting cut water loss under water deficit.

## Conclusions

Aquaporins, present in all kinds of plants tissues, control water transportation across membranes and can regulate water flow in whole plants by changing their amount and activity. In the current study, by transgenic technology, we found that ectopically expressing *MdPIP1;3* conferred enhanced drought tolerance to transgenic tomatoes partially via reduced water loss controlled by stomata closure in leaves. In addition, the transgenic tomato fruits ectopically expressing *MdPIP1;3* were larger and heavier with larger and engorged cells, which might result from more efficient water transportation across membranes in transgenic fruit cells. Our study will provide new inspirations to molecular breeding, by engineering apples with big fruits via efficient water transportation under well-watered condition and enhanced drought tolerance under water deficit, which will contribute to apple production.

## Additional file

Additional file 1: Figure S1. The expression of *SlPIP1s* and *SlPIP2s* under normal and drought conditions in wild type and transgenic tomato plants ectopically expressing *MdPIP1;3*. The semi-quantitative RT-PCR was conducted to check the expression of *SlPIP1s* (*SlPIP1;2*, Solyc03g096290.2.1; *SlPIP1;3*, Solyc08g008050.2.1; *SlIP1;5*, Solyc01g103270.2.1) and *SlPIP2s* (*SlPIP2;1*, Solyc06g011350.2.1; *SlPIP2;5*, Solyc02g083510.2.1; *SlPIP2;7*, Solyc01g111660.2.1) in the three transgenic tomato lines ectopically expressing *MdPIP1;3* and wild type seedlings before and 4 h after drought treatment, respectively. It is obviously that all the aquaporin genes were induced by drought stress. But there isn’t any difference in expression of these genes between wild type and transgenic tomato plants. (TIFF 67 kb)

## Abbreviations

CMV: Cauliflower mosaic virus
MIP: Major intrinsic protein
MS: Murashige and Skoog
ORF: Open reading frames
WT: Wild type
