# Supplementary figures and images for: Ectopically expressing MdPIP1;3, an aquaporin gene, increased fruit size and enhanced drought tolerance of transgenic tomatoes
Source: BMC Plant Biol. 2017 Dec 19;17:246. doi: 10.1186/s12870-017-1212-2 (PMC5735821; doi:10.1186/s12870-017-1212-2)

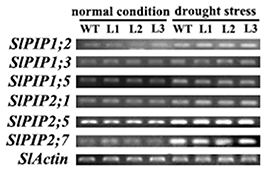

Supplement: Additional file 1: Figure S1. — The expression of SlPIP1s and SlPIP2s under normal and drought conditions in wild type and transgenic tomato plants ectopically expressing MdPIP1;3. The semi-quantitative RT-PCR was conducted to check the expression of SlPIP1s (SlPIP1;2, Solyc03g096290.2.1; SlPIP1;3, Solyc08g008050.2.1; SlIP1;5, Solyc01g103270.2.1) and SlPIP2s (SlPIP2;1, Solyc06g011350.2.1; SlPIP2;5, Solyc02g083510.2.1; SlPIP2;7, Solyc01g111660.2.1) in the three transgenic tomato lines ectopically expressing MdPIP1;3 and wild type seedlings before and 4 h after drought treatment, respectively. It is obviously that all the aquaporin genes were induced by drought stress. But there isn’t any difference in expression of these genes between wild type and transgenic tomato plants. (TIFF 67 kb) [file 12870_2017_1212_MOESM1_ESM.tif]
